# Joint Sparing: The Key to Unlocking Early Treatment Success in Eosinophilic Fasciitis

**DOI:** 10.7759/cureus.60076

**Published:** 2024-05-11

**Authors:** Tatsuki Yamada, Etaro Hashimoto, Masatsune Suzuki, Kazuhito Hirose

**Affiliations:** 1 Department of General Medicine and Primary Care, University of Tsukuba Hospital, Tsukuba, JPN; 2 Faculty of Medicine/Department of Primary Care and Medical Education, University of Tsukuba/University of Tsukuba Hospital, Tsukuba, JPN; 3 Department of Primary Care and Medical Education/Department of General Medicine and Primary Care, Faculty of Medicine, University of Tsukuba/University of Tsukuba Hospital, Tsukuba, JPN; 4 Department of General Medicine, Tsukuba Medical Center Hospital, Tsukuba, JPN

**Keywords:** eosinophilia, groove sign, orange-peel sign, diffuse fasciitis, eosinophilic fasciitis

## Abstract

Delayed diagnosis is recognized as a poor prognostic factor in eosinophilic fasciitis (EF). Elevated serum eosinophil counts, a minor criterion in the diagnostic standards, occur early in the disease course. However, signs such as the groove sign and orange-peel sign typically do not appear in the initial stages, posing challenges for early detection under the current diagnostic criteria. We report a case where the combination of "joint sparing" physical findings and elevated eosinophil counts facilitated early diagnosis and treatment. A 79-year-old woman presented with an acute onset of swelling in the upper and lower limbs. Physical examination revealed non-pitting edema with "joint sparing”, and blood tests showed increased eosinophil counts. Contrast-enhanced MRI of the lower limbs showed post-contrast enhancement along the fascia, leading to a diagnosis of EF.

The presence of non-pitting edema with "joint sparing” may be a valuable diagnostic indicator for EF. Furthermore, combining this with serum eosinophil counts can enable early diagnosis and treatment, potentially improving patient outcomes.

## Introduction

Eosinophilic fasciitis (EF) is characterized by an acute to subacute onset of symmetrical swelling of the limbs, followed by skin hardening. This condition can lead to treatment resistance if not diagnosed and managed promptly [1]. Delayed diagnosis has been identified as a poor prognostic factor, underscoring the importance of early recognition and intervention [2,3].

Currently, the diagnostic criteria proposed by Pinal-Fernandez et al. in 2014 and by Jinnin M et al. in 2018 are utilized [4,5]. Both sets of criteria suggest the presence of symmetrical plate-like sclerotic lesions on all four limbs and the exclusion of systemic sclerosis as major criteria [4,5]. Additionally, skin changes, such as erythema, swelling, and induration, are noted. Minor criteria, according to Pinal-Fernandez I et al., include the groove sign, orange-peel appearance, eosinophilia >500/μL, and elevated aldolase levels [5]. MRI of the limbs is useful in detecting these conditions, showing increased T2 signal in the subfascial and deep fascial layers and enhanced structure on fat-suppressed T1 images post-gadolinium, indicative of fascial inflammation [6]. Muscle biopsy reveals edema and inflammatory infiltrates with lymphocytes, plasmocytes, histiocytes, and predominantly eosinophils in the deep fascia and lower subcutis. Over time, the fascia thickens and becomes sclerotic, with the inflammatory infiltrate disappearing [6].

The therapeutic approach in EF remains unclear, and there are no randomized studies on therapy. Empirical treatments have been reported [7]. The initial treatment involves administering 1 mg/kg per day of Prednisone, with subsequent tapering [8]. Other immunosuppressive and immunomodulatory agents are considered to achieve a therapeutic response or to spare the use of glucocorticoids, especially in patients unresponsive to 1.5 mg/kg/day of Prednisone administered for three months [8].

Skin findings initially include non-pitting edema on the full circumference of the distal limbs (forearms and lower legs). This edema is later replaced by symmetrical induration with puckering, giving the skin an "orange peel" texture [6]. Characteristically, transient eosinophilia occurs during the acute phase [4]. Serum eosinophil counts can naturally return to normal levels, but the duration remains unclear [9,10]. Initially, patients may present with only eosinophilia and non-pitting edema, without the specific "orange peel" and symmetrical induration associated with EF. Additionally, the presence of the orange-peel sign has been linked to treatment resistance, indicating a more severe disease course [11]. This highlights a significant gap in the current diagnostic approach, where the existing criteria may not facilitate the early detection and diagnosis of EF.

The purpose of this report is to propose that "joint sparing" may be a new clinical indicator for the early diagnosis and treatment of EF. By identifying this feature at an earlier stage, we aim to improve patient outcomes through timely intervention. This report seeks to address the limitations of current diagnostic criteria by providing evidence for "joint sparing" as a reliable early sign of EF, potentially leading to a paradigm shift in the management of this condition.

## Case presentation

A 79-year-old female patient presented to our institution with an acute onset of bilateral lower limb edema 10 days prior, which subsequently extended to both upper limbs. She was referred to our institution for further evaluation after prior consultations at another medical facility failed to elucidate the etiology of her generalized acute edema, where common causes like heart failure were explored but a definitive diagnosis was not established.

**Figure 1:**
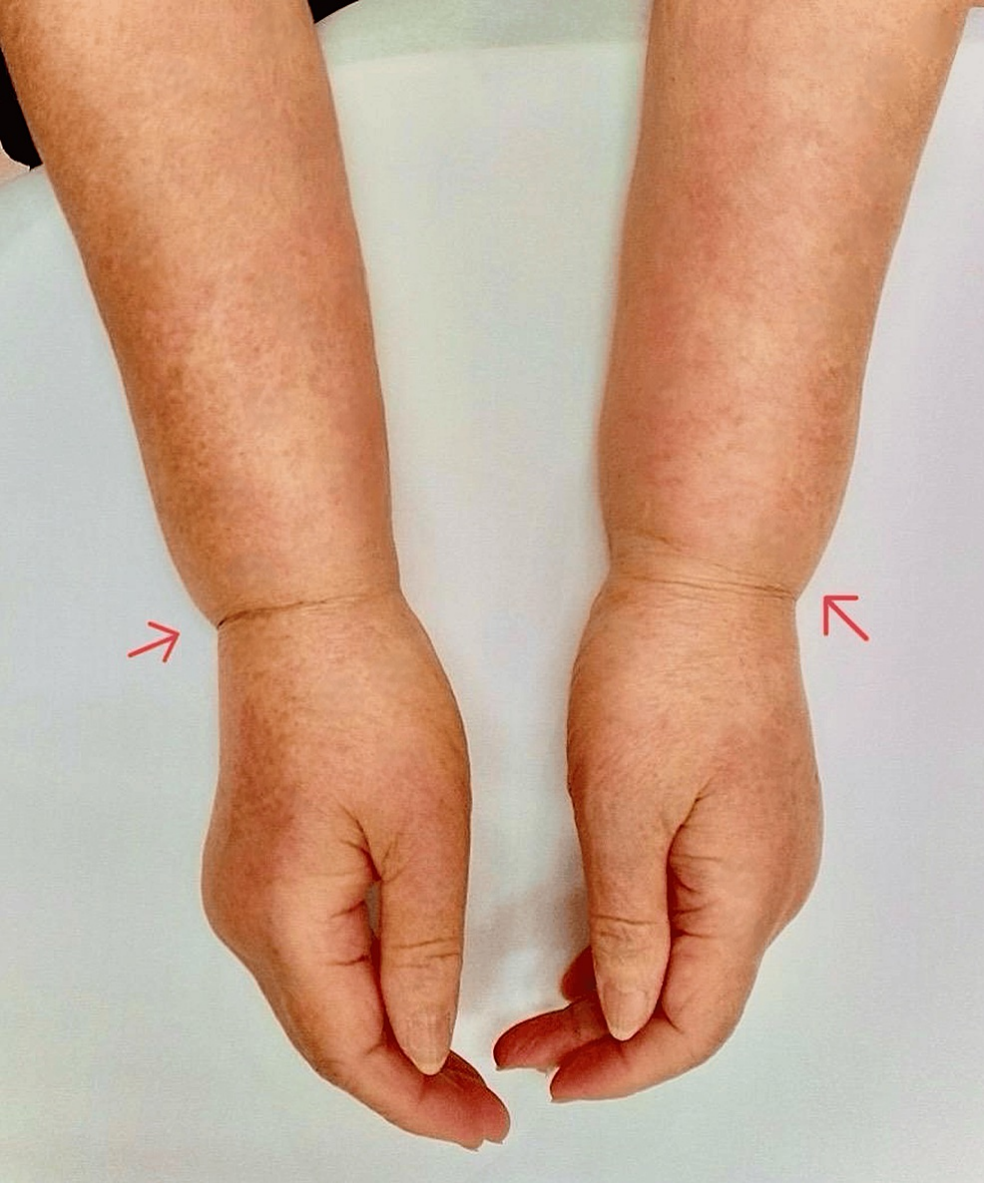
Images of bilateral forearms and hands The swelling conspicuously spared the wrist joints, a phenomenon described as "joint sparing" (arrow).

Physical examination identified non-pitting edema extending from the distal extremities, accompanied by tenderness upon palpation. Notably, the swelling conspicuously spared the wrist joints, a phenomenon described as "joint sparing” (Figure 1).

The subjects exhibited no symptoms of Raynaud's phenomenon, nor did they present with noticeable groove signs, orange-peel signs, or lesions of internal organs.

Laboratory investigations showed a normal white blood cell count, an increased absolute eosinophil count, elevated aldolase levels above the normal range, and anti-Scl-70 antibodies within the negative range (Table 1).

**Table 1:** Laboratory findings

test	Observed Value	Reference Range
Total protein	6.2 (g/dL)	6.6-8.1 (g/dL)
Albumin	3.3 (g/dL)	4.1-5.1 (g/dL)
Serum creatinine	0.69 (mg/dL)	0.46-0.79 (mg/dL)
Creatine kinase	157 (IU/L)	41-153 (IU/L)
C-reactive protein	0.27 (mg/dL)	Less than 0.14 (mg/dL)
Erythrocyte sedimentation rate	25 (mm/hr)	0-15 (mm/hr)
Immunoglobulin G	1170 (mg/dL)	861-1747 (mg/dL)
Immunoglobulin A	160 (mg/dL)	93-393 (mg/dL)
Immunoglobulin M	147 (mg/dL)	50-269 (mg/dL)
Antinuclear antibody	1:40 homogenous pattern	negative
PR3-anti-neutrophil cytoplasmic antibody	<1.0 (U/mL)	Less than 3.5 (U/mL)
MPO-anti-neutrophil cytoplasmic antibody	<1.0 (U/mL)	Less than 3.5 (U/mL)
Rheumatoid factor	20.6 (IU/mL)	Less than 15 (IU/mL)
Aldolase	7.3 (IU/L)	1.7-5.7 (IU/L)
Anti-scleroderma antibody	1.7 (U/mL)	16.0 (U/mL)
White blood cell	6,000 (/μL)	3300 (/μL)
Absolute eosinophil count	1,326 (/μL)	Less than 500 (/μL)

**Figure 2:**
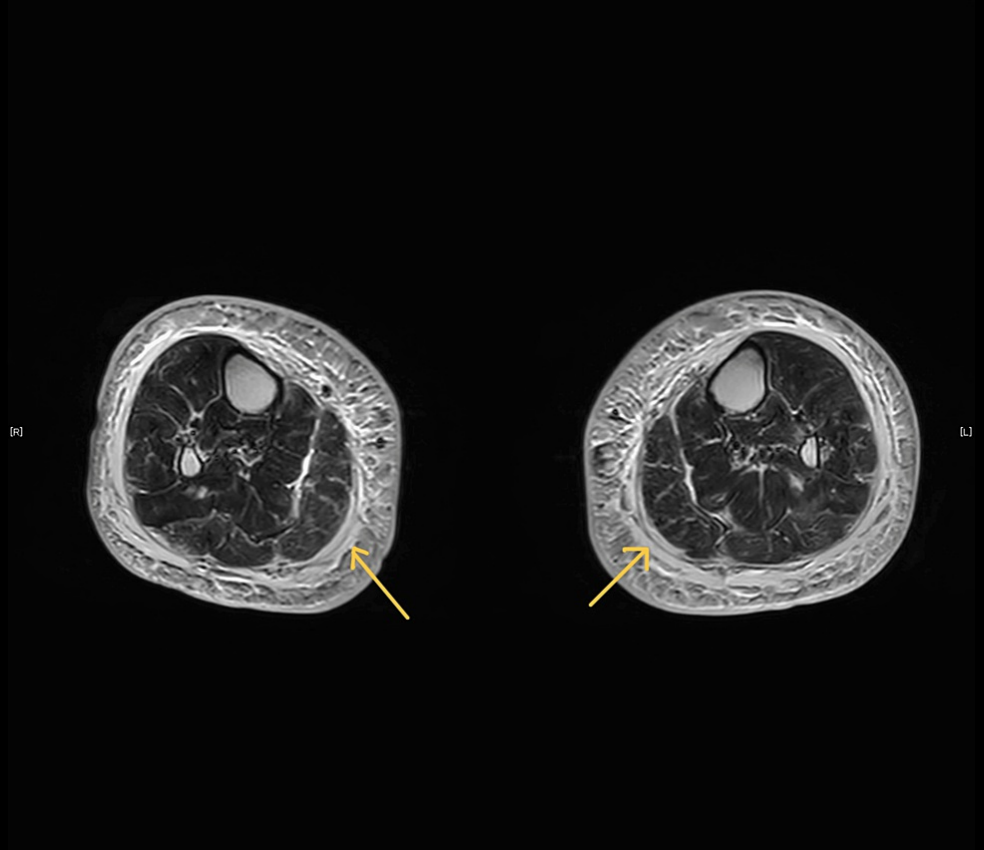
Images of the T2 signal This figure illustrates increased signal intensity in the gastrocnemius and flexor muscle groups in the lower limb, as revealed by contrast-enhanced MRI on the T2 signal (arrow).

Imaging studies, such as a contrast-enhanced CT scan of the chest and abdomen, revealed no malignancies. Lower limb contrast-enhanced MRI revealed increased signal intensity in the gastrocnemius and flexor muscle groups on the T2 signal (Figure 2).

**Figure 3:**
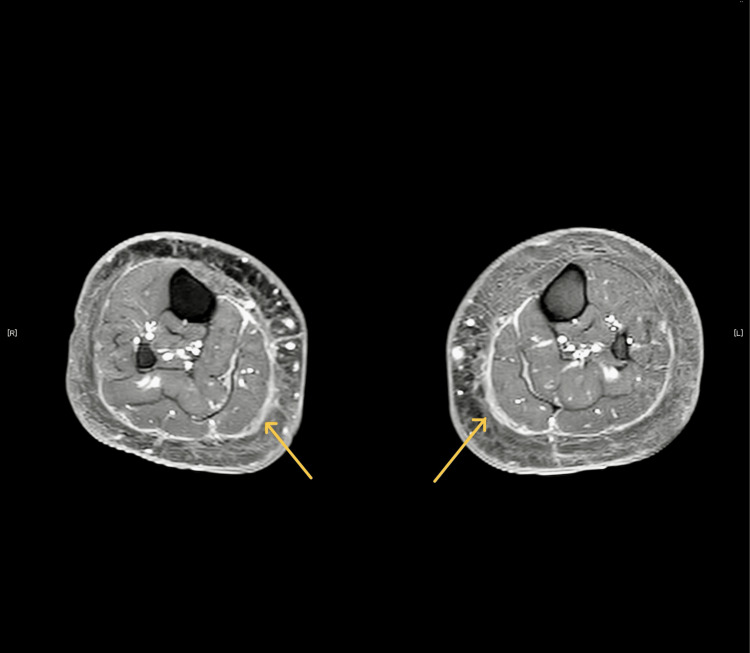
Images of fat-suppressed T1 signal This figure showcases the enhanced visualization of structures along the fascia in the lower limb following gadolinium administration, as captured on fat-suppressed T1 images (arrow).

After gadolinium administration, enhanced visualization of the structures on fat-suppressed T1 images was noted along the fascia. These findings indicate inflammation of the fascial layers (Figure 3).

A muscle biopsy from the lower leg was performed, revealing inflammatory cell infiltration around the striated muscle bundles emanating from the fascia. Although eosinophilic infiltration was sparse, the histopathological findings were consistent with eosinophilic fasciitis.

The patient was initiated on a treatment regimen of prednisolone at a dose of 50 mg/day (0.7 mg/kg). The edema improved over time, after which the dosage was reduced to 45 mg/day, and the patient was discharged to home care. One year after the start of treatment, the patient has successfully reduced the prednisolone dosage to 6 mg/day without any recurrence. 

## Discussion

We report a case of eosinophilic fasciitis where early diagnosis and treatment were achieved by focusing on "joint sparing”. Eosinophilic fasciitis (EF) is characterized by acute to subacute onset of symmetrical swelling of the limbs, followed by skin hardening, a condition that can lead to resistance to treatment if not diagnosed and treated promptly [1]. The groove sign and orange-peel sign, considered minor criteria, typically do not appear until several weeks after disease onset, limiting their utility for early diagnosis [5]. This highlights a significant gap in the current diagnostic approach, where the existing criteria may not facilitate the early detection and diagnosis of EF.

Our case report proposes two possibilities for the early diagnosis of EF. Firstly, the phenomenon of "joint sparing" could be a useful and specific clinical sign for diagnosing EF amid numerous nonspecific symptoms. Firstly, the phenomenon of "joint sparing" emerges as a potentially invaluable clinical sign in the diagnosis of EF, offering a beacon of specificity in a sea of nonspecific symptoms. Secondly, the combination of an elevated serum eosinophil count and the aforementioned "joint sparing" sign may be useful for the early detection of EF. This synergistic approach not only paves the way for timely therapeutic intervention but also portends an amelioration in patient functional prognosis. These findings, distilled from our meticulous observation, suggest a recalibration of diagnostic paradigms, potentially enhancing the precision and expediency of EF diagnosis. 

It is reported that inflammatory joint involvement is present in less than half of the patients diagnosed with EF [6]. However, joint contractures and associated functional limitations, generally resulting from the infiltration of the fascia surrounding the joints, are less likely to occur in the early stages of the disease [12]. Additionally, it is known that the initial symptoms of EF are acute to subacute swelling. Therefore, even in the absence of joint mobility restrictions, swelling due to fasciitis may occur earlier. In such cases, swelling that spares the joints is formed. "Joint sparing" refers to a condition where swelling is present without involving the joints directly, indicating that the swelling is primarily due to structures that cannot cross the joints such as fascial inflammation in the case of EF. These observations underscore the potential utility of "joint sparing" as an early diagnostic clue in EF, suggesting that its presence could significantly aid in the timely identification of this condition.

Our second finding elucidates the synergistic role of elevated serum eosinophil counts in conjunction with "joint sparing" in facilitating the early diagnosis and treatment of EF. This dual-parameter approach enhances the specificity and sensitivity of early EF detection, potentially circumventing the delays associated with traditional diagnostic methods. Literature supports that patients’ laboratory tests usually show transitory peripheral eosinophilia [4]. By integrating "joint sparing" with eosinophilia, our findings offer a more targeted diagnostic criterion, potentially distinguishing EF from other eosinophilic conditions more effectively.

By adopting a diagnostic approach that incorporates "joint sparing" and elevated eosinophil counts, clinicians can potentially identify EF at a nascent stage, thereby facilitating earlier intervention and improving patient outcomes. This approach not only underscores the importance of a thorough physical examination but also highlights the need for clinicians to be vigilant for these signs in patients presenting with unexplained skin and soft tissue symptoms. Early diagnosis and treatment initiation are paramount in preventing the progression to irreversible fibrosis, underscoring the clinical utility of our findings in enhancing patient care. Future research should investigate the frequency, sensitivity, and specificity of joint sparing, as well as further elucidate the pathophysiology.

## Conclusions

Delayed diagnosis is recognized as a poor prognostic factor in eosinophilic fasciitis (EF). However, it poses challenges for early detection under the current diagnostic criteria.

Our report underscores two main points. First, the presence of "joint sparing" may be a key early sign that can help identify eosinophilic fasciitis (EF) among many unclear symptoms. Second, when we see "joint sparing" together with high eosinophil counts in the blood, it's a strong hint that EF might be the cause. This combination offers a new way to spot EF early, which can lead to quicker treatment and better chances for the patient to get well. Our findings suggest we might need to change how we diagnose EF to catch it earlier and more accurately. This new approach could make a big difference in treating EF effectively and improving outcomes for patients. However, at this time, there is a lack of evidence to support this assertion other than our observations in this case. Future research is needed.
